# Advanced Systems Biology Methods in Drug Discovery and Translational Biomedicine

**DOI:** 10.1155/2013/742835

**Published:** 2013-09-19

**Authors:** Jun Zou, Ming-Wu Zheng, Gen Li, Zhi-Guang Su

**Affiliations:** Molecular Medicine Research Center, State Key Laboratory of Biotherapy, West China Hospital, West China School of Medicine, Sichuan University, Chengdu 610041, China

## Abstract

Systems biology is in an exponential development stage in recent years and has been widely utilized in biomedicine to better understand the molecular basis of human disease and the mechanism of drug action. Here, we discuss the fundamental concept of systems biology and its two computational methods that have been commonly used, that is, network analysis and dynamical modeling. The applications of systems biology in elucidating human disease are highlighted, consisting of human disease networks, treatment response prediction, investigation of disease mechanisms, and disease-associated gene prediction. In addition, important advances in drug discovery, to which systems biology makes significant contributions, are discussed, including drug-target networks, prediction of drug-target interactions, investigation of drug adverse effects, drug repositioning, and drug combination prediction. The systems biology methods and applications covered in this review provide a framework for addressing disease mechanism and approaching drug discovery, which will facilitate the translation of research findings into clinical benefits such as novel biomarkers and promising therapies.

## 1. Introduction

Advances in biological sciences over the past several decades have led to the generation of a large amount of omics molecular data at the level of genome, transcriptome, proteome, and metabolome. While identifying all the genes and proteins provides a catalog of individual molecular components, it is not sufficient by itself to understand the complexity inherent in biological systems. We need to know how individual components are assembled to form the structure of the biological systems, how these interacting components can produce complex system behaviors, and how changes in conditions may dynamically alter these behaviors. As a result, systems biology has emerged as an important new discipline that addresses the current challenge of interpreting the overwhelming amount of genome-scale data on a systems level.

Yet remaining in its infancy in many ways, systems biology is in an exponential development stage in recent years and has been widely used in pharmacology to better understand molecular basis of disease and mechanism of drug action [1]. It has become apparent that many diseases such as cancer are much more complex than initially anticipated, because they are often caused by a combination of multiple molecular abnormalities, which supports a novel network perspective of complex diseases [2]. In addition, many drug candidates failed clinical phases because the mechanisms of the cellular pathways they target are incompletely understood. These have significant implications in the drug discovery process because the molecular components that need to be targeted must change from single proteins to entire cellular pathways [3]. By considering the biological context of drug target, systems biology provides new opportunities to address disease mechanisms and approach drug discovery, which will facilitate the translation of preclinical discoveries into clinic benefits such as novel biomarkers and therapies [4].

In the following sections, we will first describe systems biology methods that have become commonplace; then we will examine their various applications in drug discovery and translation medicine; finally brief discussions on future directions are given.

## 2. Systems Biology Methods

Systems biology focuses on developing an understanding of how the phenotypic behavior of biological system as a whole emerges from individual molecular components and their interactions that constitute the biological system [5]. Thus a key feature of systems biology is that interactions among many components are studied, rather than simply the characteristics of individual molecules. Another feature is that systems biology uses a range of computational approaches to generate predictions that can be tested experimentally. Systems biology thus relies on a combination of experiments that measure multiple cellular components and computational approaches that allow the analysis of various data sets. As an iterative process, computational modeling is performed to propose nonintuitive hypotheses that can subsequently be experimentally validated, and the newly acquired quantitative experimental data can then be used to refine the computational model that recapitulated the biological system of interest.

In general, two complementary computational approaches are used in systems biology, namely, data-driven and hypothesis-driven methodologies (also called top-down and bottom-up modeling) [6]. The data-driven approaches involve the gathering of large-scale omics data sets and subsequent analyses of these data using statistical modeling techniques. Network modeling, one of the most frequently used data-driven approaches, provides insights into the interactions among hundreds or even thousands of molecular components. On the other hand, the hypothesis-driven approaches are generally applied to relatively small systems with fewer molecular components. A major challenge to this approach is that the quantitative details of the interactions are unknown and so it is necessary to hypothesize relevant forms of the equations that govern the interactions and estimate the values of the associated parameters [6]. Dynamical modeling, the major hypothesis-driven approach, can be employed to characterize the quantitative relations between molecular components and the emergent behaviors that arise from their interactions. Choosing the appropriate modeling approaches depends on the nature of the data and the level of understanding of the studied biological system.

## 3. Network Modeling in Biological Systems

A “network” refers to a collection of “nodes” and a collection of “edges” that connect pairs of nodes. Network representation of biological molecular systems typically considers molecular components as nodes and their interactions or relationships as edges. In biological networks, molecular components can be genes, proteins, metabolites, drugs, or even diseases and phenotypes; interactions can be direct physical interactions, metabolic coupling, and transcriptional activation. Different types of biological networks can be constructed, such as protein-protein interaction networks, cellular signaling networks, gene regulatory networks, disease gene interaction networks, and drug interaction networks [7, 8].

Network analysis of biological systems is increasingly gaining acceptance as a useful method for data integration and analysis. Assembling a network to represent the complexity of biological systems is just recognized as the beginning of the analysis. A series of advances in graph-based theory are also relied on to provide insights into the topology properties and organizational principles of biological networks, which include information about the properties of nodes and edges, global (i.e., the entire network) topological properties, hubs, motifs, and modules [2, 7]. Properties of nodes include degree (also called connectivity degree), node betweenness centrality, closeness centrality, and eigenvector centrality. Properties of edges include edge betweenness centrality, relationship types (i.e., activation or inhibition), and edge directionality. Global topological characteristics of networks include connectivity distribution, characteristic path length, clustering coefficient, grid coefficient, network diameter, and assortativity [7].

The degree of a node is the number of edges that connect to it; for example, the degree of a protein could represent the number of proteins with which it interacts. An important realization is that networks in biological systems, including protein-protein interaction and metabolic networks, are scale-free, which means that the degree distribution (i.e., the fraction of nodes with a given degree) has a power-law tail. By contrast, for a random network, most nodes have approximately the same number of edges (i.e., fits a Poisson distribution). The scale-free architecture makes biological networks robust to random failures [7].

Network motifs are recurring small subnetworks composed of a few nodes and their edges, and the topology types of these subnetworks appear in biological networks much more frequently than expected by chance [8]. Some motifs are particularly important because they are likely to be associated with some optimized biological function; examples include negative feedback loops, positive feed-forward loops, bifans, or oscillators. Another characteristic of networks is their modularity (i.e., network clustering), implying the existence of ‘modules', which are network neighbourhoods with locally dense connectivity segregated by regions of low connectivity [8]. In biological networks, a module could correspond to a group of molecules that tend to interact with each other to achieve some closely related cellular functions.

Highly connected nodes in a network are called hubs. The biological role of hubs allows for their classification into “party” hubs and “date” hubs [2]. Party hubs, also called intramodule hubs, are highly coexpressed with their interacting molecules and preferentially function inside modules. While date hubs, also called intermodule hubs, appear to be more dynamically regulated relative to their interacting molecules and preferentially link different functional modules to each other. For example, Chang et al. have recently identified modules enriched in closely connected “party hubs” that all participate in the same biological process “ribosome biogenesis and assembly” [9]. Whereas *CDC28*, predicted as a “date hub,” serves as an intermodule coordinator and performs important functions in the regulation of both “cell cycle” and “DNA damage” [9].

## 4. Dynamical Modeling in Biological Systems

Dynamical modeling, also named mechanistic modeling, can be viewed as translations of familiar pathway maps into mathematical form [10]. Equations in dynamical models, derived from established physicochemical theory (e.g., the law of mass action and Michaelis-Menten kinetics), seek to describe biomolecular processes (such as intermolecular association, catalysis and covalent modification, and intracellular localization). Kinetic parameters in dynamical models have physicochemical interpretations that define the reaction rate and binding affinity.

Provided that reasonable values for kinetic parameters and initial concentrations of cellular components can be obtained, simulation of the dynamical models yields the concentrations of each component at subsequent times, thereby facilitating comparison of simulated and experimental time courses [5]. Thus dynamical modeling uses prior knowledge to make specific predictions and works best with pathways in which components and connectivity are relatively well established. Used appropriately, dynamical modeling is much more powerful in analyzing molecular events in a cellular context, revealing the principles of biological systems, and generating novel and useful hypotheses.

The correct mathematical form for a dynamical model depends on the properties of the system being studied and the goals of the modeling effort. Ordinary differential equations (ODEs) and partial differential equations (PDEs) are the most commonly used forms. ODEs represent the rates of production and consumption of individual biomolecular component in terms of mass action kinetics, which is an empirical law stating that rates of a reaction are proportional to the concentrations of the reacting components [11]. Each biochemical transformation is therefore represented by an elementary reaction with forward and reverse rate constants. One fundamental assumption of ODEs is that the cellular compartment is well mixed; that is, the concentration of each component is high and transports instantaneously within a compartment [12]. If this assumption is not satisfied, then it is necessary to use PDEs to explicitly simulate the changes in component concentrations with respect to space. Defining a PDEs model requires assigning components and reactions to the appropriate cellular compartment where they occur, diffusion rules and constants governing the transfer of components among different compartments, and the boundary constraints of each compartment [12].

Dynamical systems can be in either deterministic or stochastic form [5]. A dynamical system is deterministic if its trajectory is uniquely determined by the initial state and a given parameter set, while a stochastic dynamical model can go to different states with different probabilities even at a given initial state. Stochastic simulations include effects arising from random fluctuation around the average behavior, such as small molecules number of given component, sufficiently low elementary reactions, or cell-to-cell variability due to intrinsic noise.

To develop a dynamical model, there are approximately four steps. (1) Model design: one of the initial stages is to specify the model scope and establish the reaction scheme of all of the molecular components of interest. This may involve a connectivity diagram listing all of the components (including their biochemically modified versions), their connections (such as stimulatory or inhibitory connections and physical interactions), and their appropriate subcellular location [12]. (2) Model construction: according to the physicochemical theory, the connectivity diagram must be converted into appropriate biochemical reactions, which are mathematically represented by differential equations [13]. Once these reactions have been established, the experimental data needed for the kinetic parameters and initial concentrations are implemented [12]. (3) Model calibration also known as model regression, is the process by which unknown kinetic parameter values in a model are estimated so as to match model performance to experimental measurements. The parameter estimation is generally based on data-fitting techniques that involve an iterative process of adjusting kinetic parameter values to minimize the difference between the model predicted value and the corresponding experimental data [14]. (4) *Model validation* is the process of evaluating the goodness of a calibrated model. This includes making predictions that can be subjected to experimental test. If the simulation results of the dynamical model recapitulate experimentally defined input-output relations, then the model can be considered to be accurate. The input-output relations may be time-course and dose-response experimental data in the presence or absence of additional perturbations [13].

For many biological systems, there are insufficient kinetic parameters for the biochemical reactions, which have posed an obstacle for the application of quantitative dynamical models based on ODEs or PDEs. To address this issue, discrete dynamical modeling has been used to provide an alternative way to qualitatively describe complex biological systems with many unknown kinetic parameters. In these models, the states of the cellular components are qualitative, and the time variable is often considered to be discrete. The main types of discrete dynamical models include Boolean networks and Petri nets [15]. Boolean networks, whose node is described by only two qualitative states (i.e., ON and OFF), have been successfully applied in modeling gene regulatory networks and signaling networks [16, 17]. Petri nets, which contain two types of nodes representing the cellular components and the biochemical reactions, are particularly suited for modeling metabolic networks and analyzing metabolic disorders [18].

## 5. Systems Biology Methods to Human Disease

Compared with traditional reductionist approach that attempts to explain complex diseases by studying individual gene, systems biology is characterized by the view that the underlying mechanism of complex diseases is likely to be the dysregulation of multiple interconnected cellular pathways. Therefore, biological network analyses and dynamical modeling have been increasingly used to underlie the genotype-phenotype relationships in human disease [8]. Here, we attempt to cover four recent advances in this area: (1) studies of global relationships between human disease and associated genes, (2) predictions of treatment response, (3) investigations of the underlying mechanism of diseases, and (4) predictions of new disease-associated genes.

### 5.1. Human Disease Networks

 Most previous studies have focused on the association between a single gene and a single disease, whereas systems biology approaches using network-based tools enable a better understanding of the relationships among multiple genes and diseases. Goh et al. used the collected gene-disease associations to build the first human disease network by linking diseases that share one or more disease genes, and it shows that similar pathophenotypes have a higher likelihood of sharing genes than do pathophenotypes that belong to different disease classes [19]. They also found that most disease genes are nonessential and are not encoded by hub proteins. Linghu et al. explored the relationships between diverse diseases and disclosed hidden associations between disease pairs having dissimilar phenotypes [20]. Suthram et al. present an integrated network approach to identify significant similarities between diseases and reveal common disease-state modules significantly enriched for drug targets [21]. Such systematic approaches have also provided a foundation for a genome-scale network analysis of complex diseases, such as cancer [22], neurodegenerative disease [23], inflammatory disease [24], and also pathogen responses [25].

### 5.2. Treatment Response Prediction

 An important area in which systems biology approaches have been applied is biomarker discovery. Several groups have begun to integrate gene and protein expression profiles with system-wide maps of the pathways to identify biomarkers able to diagnose disease severity and predict disease outcomes. A recent study illustrated how the network-based approach that identifies subnetworks with coherent expression patterns can be used to identify novel markers for breast cancer metastasis [26]. The subnetwork-based analysis of gene expression profiles has also successfully been used to predict the relative risk for disease progression and patient survivability [27–30]. In all cases, the goal is to identify biomarkers not as lists of individual genes or proteins but as functionally related groups of genes or proteins whose aggregate properties account for the phenotypic differences between the different populations of patients [31]. Unlike conventional expression diagnostic markers based on individual genes, these network-based diagnostic markers should be inherently more reliable since they provide the biological interpretation for the association between the subnetwork biomarker and the particular type of disease [32].

### 5.3. Investigation of Disease Mechanisms

 Based on the construction of gene regulatory networks from large-scale molecular profiles, systems biology approaches have been valuable for elucidating the mechanisms of both physiological regulation [33] and pathological processes in complex diseases [32]. Recent observations have shown that the wiring of biological networks can change from healthy to diseased states. For instance, inflammatory and immune signaling pathways show different functional wiring when comparing normal and transformed hepatocytes [34]. Rozenblatt-Rosen et al. systematically examined host interactome and transcriptome network perturbations caused by DNA tumor virus proteins [35]. The resulting integrated viral perturbation data reflects rewiring of the host biological networks and highlights pathways, such as Notch signaling and apoptosis, that go wrong in cancer. Zhang et al. constructed gene regulatory networks to characterize molecular systems associated with Alzheimer's disease [36]. Their network-based integrative analysis not only highlighted the strong association of immune pathways with the pathophysiology of the disease but also identified the key network regulators that may serve as effective targets for therapeutic intervention. Another thrust in systems biology involves combining dynamical modeling of regulatory pathways with molecular and cellular experiments as a means to understand the precise regulatory mechanisms of networks that are altered in diseases [37–39].

### 5.4. Disease-Associated Gene Prediction

The search for disease-causing genes is a long-standing goal of biomedical researches. Systems biology is playing an increasing role in this area through the computational integration of multiple genome-scale measurements. It is assumed that if biological networks underlie genotype-phenotype relationships, then network properties should be able to predict unidentified human disease-associated genes. In an early example, network modeling strategies have been successfully used in tumor research. Starting with known genes encoding tumor suppressors of breast cancer, Pujana et al. generate a network containing genes linked by potential functional associations, and the analysis of this network permitted identification of novel genes potentially associated with higher risk of breast cancer [40]. Mani et al. introduce a systems biology approach, based on the analysis of the network of molecular interactions that become dysregulated in specific tumors, to decipher the human B-lymphocyte interactome, which helped to identify causal oncogenic lesions in several B-cell lymphomas [41]. Similar network-based computational frameworks have been proposed to reliably predict disease-associated genes [42, 43]. It is thus suggested that studying dysregulation at a biological network level, rather than in a “gene centric” manner, can provide a highly efficient method for addressing the problems of identifications of genes playing a role in human disease.

## 6. Systems Biology Methods in Drug Discovery

Systems biology approaches have long been used in pharmacology to understand drug action. The application of computational and experimental systems biology methods to pharmacology allows us to introduce the definition of “systems pharmacology” [44], which describes a field of research that provides us with a comprehensive view of drug action rooted in molecular interactions between drugs and their targets in a human cellular context. Advances in systems pharmacology will, in the long term, assist in the development of new drugs and more effective therapies for patient treatment management. There are several important clinically motivated applications in drug discovery to which systems biology approaches make significant contributions. Here, we attempt to discuss five recent advances in this field: (1) drug-target networks, (2) predictions of drug-target interactions, (3) investigations of the adverse effects of drugs, (4) drug repositioning, and (5) predictions of drug combination.

### 6.1. Drug-Target Networks

 Analysis of drug-target networks in a systematic manner shows a rich pattern of interactions among drugs and their targets in which drugs often bind to multiple rather than single molecular targets—a phenomenon known as “polypharmacology” [45, 46]. Topological analyses of this network quantitatively showed an overabundance of “follow-on” drugs, that is, drugs that target already-targeted proteins. Likewise, many proteins are targeted by more than one drug containing distinct chemical structures. This new appreciation of the role of polypharmacology has significant implications for drug development. Although the single-target approach remains the main strategy presently, some remarkable efforts are being put into the development of “promiscuous” drugs (also called “dirty drugs”) that can bind to multiple targets.

Integrating systems biology and polypharmacology holds the promise of expanding the current opportunities to improve clinical efficacy and decrease side effects and toxicity. Advances in these areas are creating the foundation of the next paradigm in drug discovery, that is, “network pharmacology” [47]. Keiser et al. related receptors to each other quantitatively based on the chemical similarity among their ligands [48]. They have shown that targets that have no obvious sequence or structure similarity are linked quantitatively based on their bioactive ligands. The unexpected relationships between drug targets could be used to predict their biological function. Li et al. developed a computational framework to build disease-specific drug-protein network, which can help study molecular signature differences between different classes of drugs in specific disease contexts [49].

### 6.2. Predictions of Drug-Target Interactions

 In recent years, the observation of polypharmacology that drugs often bind to more than one molecular target has gained attention. To fully understand the actions of a drug, knowledge of its polypharmacology is clearly essential. Keiser et al. report a computational tool that generates predictions of the pharmacological profile of drugs [50]. Unlike conventional approaches based on sequence or structural similarity between targets, the “similarity ensemble approach” defines each target by its set of known ligands, searches for drugs with chemical structure similar to the known ligands, and then predicts new drug-target associations. Campillos et al. used phenotypic side-effect similarities to infer whether two drugs share a therapeutic target. Applied to marketed drugs, a network of side-effect-driven drug-drug relations became apparent. Several unexpected drug-drug relations are formed by chemically dissimilar drugs from different therapeutic indications, which implies new drug-target relations [51]. Integrating side-effect and pharmacogenomic similarities, Takarabe et al. made a comprehensive prediction and suggested many potential drug-target interactions that were not predicted by previous approaches [52]. Cheng et al. compared three supervised inference methods and found that network-based inference performed best on prediction of drug-target interactions [53].

### 6.3. Investigations of Drug Adverse Effects

Accurate prediction of the safety and toxicology of drugs in the early stage of drug development pipelines is one of the major challenges in the pharmaceutical industry. Integrating biological data and systems biology approaches could introduce a fundamental change in the way drug candidates are assessed. Lounkine et al. use a similarity ensemble approach, which calculates whether a drug will bind to a target based on the chemical features it shares with those of known ligands, to predict the activity of marketed drugs on unintended “side-effect” targets [54]. Approximately half of their predictions were confirmed by experimental assays. An association metric was developed to prioritize those new off-targets that explained side effects better than any known target of a given drug, creating a drug-target-adverse drug reaction network. Kuhn et al. recently report a large-scale analysis to systematically predict and characterize proteins that cause drug side effects [55]. They integrated clinical phenotypic data with known drug-target interactions to identify overrepresented protein-side effect relations. The results show that a large fraction of complex drug side effects are mediated by individual proteins. Yang et al. have constructed an *in silico* chemical-protein interactome, which mimics the interactions between drugs known to cause at least one type of serious adverse effects and a panel of human proteins [56, 57]. It is revealed that drugs sharing the same adverse effects possess similarities in their chemical-protein interactome profiles. By investigating the associations between drug and off-targets, their research has explored the molecular basis of several adverse events. Other studies that integrate systems biology with structural or chemoinformatics analysis have also been conducted to successfully predict drug adverse effects [58, 59].

### 6.4. Drug Repositioning

 Drug repositioning, also called drug repurposing, is a potential alternative for drug discovery by identifying new therapeutic applications for existing drugs. The main advantage of drug repositioning is that it should drastically reduce the risks of drug development and facilitate repositioned drugs to enter clinical phases more rapidly. As one example of this utility, Iorio et al. developed an approach that exploits similarity in molecular activity signatures of all drugs to compute pair-wise similarities in drug effect and mode of action [60]. Drugs were organized into a network using the resulting similarity scores. Network theory was then applied to partition drugs into groups of densely interconnected nodes (i.e., communities). The resulting drug communities are significantly enriched for compounds with similar mode of action, which often shared the same targets and pathways. Through this approach, drug repositioning is revealed by colocation of drugs within the network communities, which predicts a shared molecular activity with other drugs in the drug communities. Gottlieb et al. proposed “PREDICT” algorithm that can handle both approved drugs and novel compounds [61]. This new method is based on the observation that similar drugs are indicated for similar diseases and utilizes the chemical similarity of drugs and disease-disease similarity measures for the prediction of novel drug indications. Furthermore, numerous systems biology approaches based on gene expression data for *in silico* drug repositioning have been published [62, 63]. Iskar et al. identified a large set of drug-induced transcriptional modules from genome-wide microarray data of drug-treated human cell lines [64]. The identified modules reveal the conservation of transcriptional responses towards drugs, thereby providing a starting point for drug repositioning.

### 6.5. Predictions of Drug Combination

 Combination therapies, which modulate multiple targets simultaneously, are essential to achieve greater therapeutic benefit than using a single drug [65]. Systems biology methods have been applied to explain and predict potential drug combinations [66]. Computational approaches utilizing dynamical modeling have already been used to simulate the effect of drug combinations and generate experimentally testable interventions [67, 68]. But due to the incomplete knowledge about the kinetic values of biochemical reactions, these dynamical models are currently restricted to a small scale and only suitable for investigating the action mechanisms of drug combination. Considering target information which is usually accessible, the combination effect of drugs might be evaluated by analyzing the interaction pattern of their targets from a network perspective [69]. Lehár et al. used large-scale simulations of bacterial metabolism to simulate the inhibitory effects of drug combinations and provided evidence that synergistic combinations are generally more specific to particular cellular phenotypes than are single agents [70]. Kwong et al. recently explored a gated signaling model that offers a new framework to identify nonobvious synergistic drug combination in NRAS-mutant melanomas [71]. Lee et al. reveal how the progressive rewiring of oncogenic signaling networks over time following EGFR inhibition leaves breast tumors vulnerable to a second and later hit with DNA-damaging drugs, demonstrating that time- and order-dependent drug combinations can be more efficacious in killing cancer cells [72].

## 7. Perspective

Systems biology is dramatically advancing our mechanistic understanding of disease progression and the discovery of novel therapeutics. Its continued success will depend on critical progress in both experimental and computational techniques. No single technique is sufficient to uncover the whole spectrum of gene-disease and drug-target relations in the context of biological systems. The main challenges that systems biology will confront over the next decade are the incompleteness of the available interactome data and the limitation of the existing computational tools. Our vision is that integrating the interactome with genome, transcriptome, proteome, and metabolome might offer a direction for the future advance of systems biology. New methodologies are also required to integrate diverse tools from systems biology, heterogeneous omics studies, chemoinformatics and bioinformatics. An integrated network that completely describes the underlying global paradigm of a cellular network should provide us with a deeper understanding of biological system. Clearly, there is much to do before systems biology can adequately demonstrate its usefulness in drug discovery and translational biomedicine, but the examples discussed here have provided a glimpse of the potential of systems biology.
